# Clinical and Biochemical Characteristics in PCOS Women With Menstrual Abnormalities 

**Published:** 2016-12

**Authors:** Vasiliki Christodoulopoulou, Eftihios Trakakis, Vasilios Pergialiotis, Melpomeni Peppa, Charalampos Chrelias, Dimitrios Kassanos, Nikolaos Papantoniou

**Affiliations:** 1 3rd Department of Obstetrics and Gynaecology, Attiko Hospital, National and Kapodistrian University of Athens, Athens, Greece; 2 2nd Department of Internal Medicine, Research Institute and Diabetes Center, Athens University Medical School, Attikon University Hospital, Athens, Greece

**Keywords:** PCOS, Anovulation, Menstrual Disorders, Glucose Intolerance

## Abstract

**Objective:** The purpose of the present study was to examine the impact of menstrual cycle abnormalities among patients with polycystic ovary syndrome (PCOS) on biochemical and anthropometric characteristics.

**Materials and methods:** We conducted a prospective observational study of patients 17-35 years of age with PCOS that attended the department of Gynecological Endocrinology of our hospital.

**Results:** A total of 309 women with PCOS participated in the study. In total, 72.2% suffered from menstrual cycle disorders. In our study 15.1% of women were overweight and 24% were obese. Also, 36% of the sample had androgenetic alopecia and 56.4% had acne. According to the stepwise discriminant analysis, we observed that glucose displayed the strongest association to the menstrual status (F to eliminate = 14.13), followed by endometrial thickness (F to eliminate = 10.89), waist circumference (F to eliminate = 10.17), LH levels (F to eliminate = 8.15) and PRL (F to eliminate = 4.45). Significantly higher levels of LH and TSH and lower levels of prolactin were found in women with menstrual disorders compared to those with normal menstrual cycles. Fasting glucose was also considerably higher among these patients although markers of insulin resistance such as the Matsuda, Quicki and HOMA-IR indices did not differ.

**Conclusion:** According to the findings of our study PCOS patients with menstrual disorders exhibit hormonal alterations and elevated fasting glucose. Future studies are needed in this field to corroborate our findings and determine the anthropometric and biochemical profile of patients with menstrual cycle irregularities.

## Introduction

Polycystic ovarian syndrome (PCOS) affects 5-12% of women of reproductive age (1). The relation of the syndrome to menstrual cycle abnormalities has been already described and it seems that menstrual irregularities seem to precede the presence of PCOS (2, 3). Current evidence supports a close relation between the degree of cycle irregularities and the grade of endocrine and metabolic disorders among these women (4, 5, 6). Hyperandrogenism has been previously described as a significant metabolic risk factor in women. Specifically, Shaw et al reported that post-menopausal women who had a history of menstrual abnormalities and elevated androgen levels had increased odds of developing coronary arterial disease and of cardiovascular events which could ultimately result to death compared with a control population (7). Obesity also seems to be significantly associated with menstrual function whereas, weight loss results in significant improvement of menstrual cycle patterns (8, 9). According to Strowitzki et al normocyclic PCOS patients have significantly better metabolic (BMI, fasting insulin, HOMA-IR) and hormonal (LH, FSH, FAI and testosterone) parameters (4). 

To date, however, we do not know the exact reasons which lead a certain percentage of PCOS patients to menstrual cycle abnormalities. This is why there seems to be a lack of consensus in this field and further studies are needed to corroborate the aforementioned findings. In the present prospective study we sought to evaluate the endocrine and metabolic differences among normocyclic and oligo-amenorheic women who suffered from PCOS in order to describe the potential predictors of menstrual abnormalities.

## Materials and methods

***Study population:*** We conducted a prospective observational study of patients 17-35 years of age with PCOS that attended the department of Gynecological Endocrinology – Child and Adolescent Gynecology at Attikon University Hospital between January 2007 and December 2015. The study was approved by the Institution Ethics Committee and is in agreement with the 1964 Declaration of Helsinki and its later amendments. After baseline assessment 321 patients who met the Rotterdam ESHRE/ ASRM – Sponsored PCOS criteria were included in the study (10).

***Anthropometric Data:*** Anthropometric measurements were obtained using classical methods. Briefly, all women were dressed in light clothes during weight measurement. For height measurement they were placed in the Frankfurt plane. Height was measured in centimeters and weight in kilograms. Waist circumference was measured using a soft tape around the 12^th^ rib, hip circumference around the iliac crest and the waist to hip ratio was calculated dividing the two measurements. Body Mass Index (BMI) was calculated as the ratio of weight/height^2^ (kg/m^2^). Classification of women as normal-weight, overweight and obese met the criteria defined by the World Health Organization. Briefly a BMI cut-off between 18.50 and 24.99 was defined as normal weight. Women below this values were classified as underweight. Overweight women were defined as those with a BMI between 25 and 29.99. Women exceeding the upper cut-off value were classified as obese.

Phenotypic characteristics were also recorded. Specifically the degree of hirsutism was assessed with the Ferriman-Gallwey- Lorenzo (FGL) index which stratifies hair growth from 0 to 4 (extensive hair growth) in each of nine locations of the body, resulting in a score that varies from a minimum of 0 up to a maximum score of 36 (11). For the study population, a score of 6 or higher was regarded as indicative of androgen excess. 

***Blood Chemistry:*** Blood was collected after an overnight fasting at 8 a.m. The HOMA insulin resistance (HOMA-IR) index was calculated from the formula: HOMA-IR Index = [(Fasting Insulin) × (Fasting Glucose)]/161, with insulin concentration expressed in pmoles/l and glucose in mmoles/l.

The quantitative insulin sensitivity check index (QUICKI) was calculated using the formula QUICKI index = 1 / (log(fasting insulin) + log(fasting glucose)), with insulin concentration expressed in pmoles/l and glucose in mmoles/l.

The Matsuda index of insulin sensitivity was calculated as follows: Matsuda index = 10,000/(G_0_ X I_0_ X G_x_ X I_x_)^0.5^ with glucose and insulin values in mmol/L and pmol/L, respectively.

We also evaluated the Lipid Accumulation Product (LAP) as follows LAP = (Waist circumference (cm)−58)×(Triglyceride concentration (mmol/l)).

***Ultrasonographic assessment:*** A transvaginal ultrasound examination was performed between day 6 – 8 of the menstrual cycle to assess ovarian morphology for the diagnosis of PCOS.

***Assays:*** Testosterone, DHEAS and cortisol measurements were performed with the analysis of Elecsys 1010/2020 and Modstar analytics E 170 by Roche with CV of 5.6%, 6% and 7%, respectively. Δ4-A, 17-OHP and FT measurements were performed with RIA kits provided by the Diagnostic Setters International Inc, Corpotage Headquarters and Medical Center Blvd, Webster Texas 77598, 4217 USA, with CV of 6.3%, 9.7% and 9.75, respectively. Glucose measurements were performed by the colorimetric method for quantitative measurement of glucose concentration in serum, plasma and urine, by Menarini diagnostics, Via Setta Santi 3, Firenze Italy, with coefficients of variance (CV) of 2.33%. Measurements of T3, T4, TSH and PRL were performed by the ADVIA Centaur system with CV of 3.44%, 5.55%, 5.87% and 4.8%, respectively. Hormone measurements were performed by the ADVIA Centaur system for FSH, LH and insulin with CV of 3.9%, 2.7% and 7.5% respectively. Activated partial thromboplastin time (APTT) was measured by clotting assay using a SIEMENS BCSC XP System Analyzer.

***Statistical analysis:*** Continuous variables are presented with mean and standard deviation (SD). The normality of the distributions was assessed with Kolmogorov-Smirnov’s test and graphical methods. Quantitative variables are presented with absolute and relative frequencies. Student’s t-test or nonparametric Mann-Whitney test were used to compare means between groups. For the comparisons of proportions chi-square and Fisher’s exact tests were used. A stepwise discriminant analysis was applied to the set of variables that significantly differed in univariate analysis. The final analysis indicated the variables with the greatest contribution in discriminating between the two groups, and finally,the discriminant model was validated by checking the percentage of group cases correctly classified after cross-tabulation of actual and predicted group membership provided by the discriminant function. All reported p values are two-tailed. Statistical significance was set at p < 0.05. All analyses were conducted using the SPSS statistical software (SPSS Inc. Released 2009. PASW Statistics for Windows, Version 18.0. Chicago: SPSS Inc.).

## Results

A total of 309 women with PCOS participated in the study. Table 1 summarizes the basic characteristics of enrolled women. In total, 72.2% suffered from menstrual cycle disorders. Among them, 58.3% of women had a cycle that exceeded 35 days, 5.2% had a cycle which lasted less than 26 days and 8.7% suffered from amenorrhea. In our study 15.1% of women were overweight and 24% were obese. Also, 36% of the sample had androgenetic alopecia and 56.4% had acne. The mean waist circumference was 90.4 ± 15.9 cm. Women with menstrual disorders had significant higher BMI and the proportion of obese women was greater in those with menstrual disorders. Additionally, women with menstrual disorders had greater mean waist and hip circumference.

**Table 1:** Sample characteristics in total and according to the existence of menstrual disorders

	Total (n = 309)	Menstrual disorders		P
		No (n = 86)	Yes (n = 223)	
	n (%)	n (%)	n (%)	
**Age, mean (SD) **	**24.9 (5.6)**	**25.4 (5.9)**	**24.6 (5.6)**	**0.260** **
**ΒΜΙ, mean (SD) **	**25.5 (6.7)**	**23.8 (4.8)**	**26.1 (7.2)**	**0.006** **
**ΒΜΙ**				
**Underweighted/ Normal**	**185 (60.8)**	**60 (70.6)**	**125 (57.1)**	**0.001** *
**Overweighted**	**46 (15.1)**	**17 (20)**	**29 (13.2)**	
**Obese**	**73 (24** **.0** **)**	**8 (9.4)**	**65 (29.7)**	
**Acne**				
**No**	**133 (43.6)**	**36 (43.4)**	**97 (43.7)**	**0.960** *
**Yes**	**172 (56.4)**	**47 (56.6)**	**125 (56.3)**	
**Androgenic alopecia**				
**No**	**187 (64** **.0** **)**	**56 (70.9)**	**131 (61.5)**	**0.138** *
**Yes**	**105 (36** **.0** **)**	**23 (29.1)**	**82 (38.5)**	
**Smoking**				
**No**	**213 (70.5)**	**61 (71.8)**	**152 (70)**	**0.768** *
**Yes**	**89 (29.5)**	**24 (28.2)**	**65 (30)**	
**Waist circumference** ** (cm)** **, mean (SD) **	**90.4 (15.9)**	**86.0 (12.4)**	**92.0 (16.7)**	**0.007** **
**Hip circumference (cm), mean (SD) **	**79.4 (28.8)**	**72.4 (22.6)**	**81.9 (30.4)**	**0.017** **

* Pearson's chi-square test;

** Student's t-test

**Table 2:** Hormone measures of women with PCOS according to the existence of menstrual disorders

	Menstrual disorders		P
	No	Yes	
	Mean ± SD	Mean ± SD	
**FSH (m/U/ml)**	**6** **.0** ** ±** **2.2**	**5.9 ±** **2.2**	**0.486** *
**LH (m/U/ml)**	**5.1 ±** **2.3**	**6.9 ±** **4.3**	**0.001** *
**PRL**	**22.7 ±** **7.5**	**16.5 ±** **9.9**	**0.002** *
**E2 (pg/ml)**	**57.4 ** **±** ** 81.3**	**49.9 ** **±** **51.3**	**0.335** *
**Testosterone (total) (ng/dl)**	**61.4 ** **±** **25.1**	**57.1 ** **±** ** 24.1**	**0.181** **
**Testosterone (free) (** **pg/ml)**	**2.14 ±** **1.34**	**2.18 ±** **1.44**	**0.717** *
**OHP17 (ng/ml)**	**1.49 ±** **1.00**	**1.25 ±** **0.75**	**0.145** *
**DHEAS (μg/dl)**	**226 ±** **119.6**	**243.3 ±** **228.3**	**0.999** *
**Δ4 ** **Androstendione** ** (ng/mg)**	**2.6** **0** ** ±** **1.30**	**2.65 ±** **1.29**	**0.606** *
**SHBG (nmol/L)**	**51 ±** **28.1**	**48.4 ±** **36.6**	**0.197** *
**Cortisole (mg/dl)**	**25.2 ±** **37.4**	**24.7 ±** **34.7**	**0.736** *
**T3 (nmol/L)**	**1.47 ±** **0.51**	**1.53 ±** **0.47**	**0.368** *
**T4 (μg/dl)**	**8.8 ±** **10.0**	**8.5 ± ** **9.1**	**0.497** *
**TSH (mU/L)**	**2.04 ±** **1.41**	**2.39 ±** **1.63**	**0.045** *
**Antithyroid antibodies**			
**Negative**	**15 ±** **93.8**	**35 ±** **83.3**	**0.423** ‡
**Positive**	**1 ±** **6.3**	**7 ±** **16.7**	
**ΙFG**			
**No**	**54 ±** **96.4**	**135 ±** **93.8**	**0.731** ‡
**Yes**	**2 ±** **3.6**	**9 ±** **6.3**	
**IGT**			
**No**	**41 ±** **95.3**	**98 ±** **89.9**	**0.353** ‡
**Yes**	**2 ±** **4.7**	**11 ±** **10.1**	

* Mann-Whitney test;

** Student's t-test;

‡ Fisher's exact test

Table 2 summarizes the hormonal characteristics of women with PCOS stratified by the presence of cycle disorders. Significantly higher levels of LH and TSH were found in women with menstrual disorders compared to those with normal menstrual cycles. Prolactin levels were also considerably lower in women with irregular menstrual cycle. The comparison of biochemical markers revealed that women with irregular menstrual cycle had higher glucose values (Table 3). None of the markers of insulin resistance and glucose regulation (Matsuda, Quicki, HOMA-IR or LAP) was significantly different in the group with menstrual disorders. This was not the case, however, when the lipid profile and the blood clotting parameters were evaluated. The endometrial thickness of women with menstrual disorders was significantly thinner (Table 4). Ovarian volume, on the other hand, was not associated with the presence of menstrual disorders.

After conducting the stepwise discriminant analysis, we observed that glucose displayed the strongest association to the menstrual status (F to eliminate = 14.13), followed by endometrial thickness (F to eliminate = 10.89), waist circumference (F to eliminate = 10.17), LH levels (F to eliminate = 8.15) and PRL (F to eliminate = 4.45). The remaining variables [BMI, waist circumference (WC) and TSH] were rejected by the analysis. The discriminant function was significant (Wilks' Lambda = 0.77, p < 0.001) indicating that the selected variables differentiate well between women with and without menstrual disorders and 74.9% of original grouped cases correctly classified.

## Discussion

In our study we aimed to identify whether menstrual cycle abnormalities result in alterations of anthropometric and biochemical parameters of PCOS patients. We observed that women with menstrual abnormalities were more likely to be obese and have an increased waist to hip ratio compared with normocyclic PCOS patients.

**Table 3:** Comparison of biochemical indices according to the presence of menstrual disorders

	**Menstrual disorders**		**P**
	**No**	**Yes**	
	**Mean ±** **SD**	**Mean ±** **SD**	
Glucose (mg/dl)	77.8 ± 11.9	83.5 ± 13.1	0.001*
Insuline (μ/U/ml)	13.8 ± 17.9	13.6 ± 10.1	0.415**
Total cholesterol (mg/dl)	176.1 ± 34.2	182.3 ± 32.4	0.201*
HDL (mg/dl)	58.1 ± 14.0	56.9 ± 21.0	0.285**
LDL (mg/dl)	110.7 ± 34.6	110.7 ± 33.5	0.991**
TG (mg/dl)	96.8 ± 71.7	94.7 ± 70.7	0.696**
Lipids (total)	496.3 ± 68.4	485.9 ± 113.9	0.713*
Lpa	11.8 ± 11.1	43 ± 86.9	0.169**
APO-a1 (mg/dl)	130.1 ± 39.3	134.5 ± 27.3	0.465*
APO-b (mg/dl)	73.7 ± 29.3	78.2 ± 21.4	0.374**
PT	15.0 ± 16.6	13.2 ± 6.0	0.669**
APTT	33.9 ± 10.6	33.7 ± 9.0	0.954**
INR	1.06 ± 0.12	1.07 ± 0.14	0.641**
CRP	3.2 ± 3.5	3.5 ± 5.2	0.681**
Adiponectine	13.7 ± 5.4	11.7 ± 5.7	0.313*
Resistine	6.2 ± 2.5	6.5 ± 1.9	0.511*
HIRSUTISM INDEX (LORENZO)	10.1 ± 4.1	9.5 ± 4	0.224**
HOMA-IR score	2.8 ± 4.0	2.7 ± 2.0	0.228**
QUICKI score	0.35 ± 0.03	0.35 ± 0.04	0.572*
MATSUDA score	10.7 ± 6.2	9.8 ± 7.2	0.140**

* Mann-Whitney test;

** Student's t-test

**Table 4:** Comparison of gynecological ultrasound measures according to the presence of menstrual disorders

	**Menstrual disorders**		**P**
	**No**	**Yes**	
	**Mean ±** **SD**	**Mean ±** **SD**	
Endometrial thickness	7.1 ± 6.8	5.2 ± 2.2	< 0.001
Ovary volume (left)	10.2 ± 3.6	10.6 ± 5.3	0.686*
Ovary volume (right)	11.1 ± 4.3	11 ± 5.1	0.595*

**Student's t-test

* Mann-Whitney test;

Fasting serum glucose was also slightly higher, although inside the normal range, among women with menstrual abnormalities. Serum insulin, HOMA-IR, QUICKI and MATSUDA indices were similar among the two groups. In our study we also examined whether the lipid accumulation product (LAP) differed among women with menstrual cycle abnormalities and those with normal cycles. Previous studies suggested that LAP is an improved marker for abnormal glucose regulation compared to BMI (12). LAP is a highly diagnostic index which might serve as a prerequisite for diagnosis of metabolic and cardiovascular diseases in PCOS women (13). According to our findings LAP did not differ among PCOS patients with menstrual cycle irregularities and normocyclic ones. 

In our study the serum lipid profile was normal in both groups. Menopausal women with a history of PCOS are at increased risk for cardiovascular events including stroke, and coronary heart disease (14). To date, it remains unclear whether the cardiovascular risk increases with the presence of menstrual cycle abnormalities among these women. The contribution of serum adiponectin and resistin as biomarkers of insulin resistance and development of the metabolic syndrome among women with PCOS has been a matter of debate during the last years (15, 16, 17, 18). In our study we investigated whether these two markers might correlate with the presence of menstrual cycle abnormalities. However, both groups of PCOS women had comparable levels of serum adiponectin and resistin. 

The hormonal profile (with the exception of LH and PRL) was also similar among the two groups, suggesting that sex hormones are not particularly altered in PCOS patients with irregular cycles. These results are in direct conflict to those of smaller previous studies (4, 19). 

The endometrial thickness during the proliferative phase significantly differed among the two groups. However, the difference was not clinically significant as it was very small (mean difference 2.1mm). Panidis et al previously suggested that the uterine volume and the endometrial thickness are smaller in women with the classic PCOS phenotype (oligo-anovulation and hyperadrogenemia) (20). To date, however, it remains unknown whether menstrual irregularities affect the endometrial environment. Hence, the institution of measurement of endometrial thickness in current clinical practice should not be taken into consideration until further evidence becomes available in the field.

## Conclusion

Current evidence do not support the hypothesis that menstrual cycle abnormalities might influence the metabolic and hormonal profile of PCOS patients. The findings of our study suggest that glucose levels, endometrial thickness, waist circumference and serum LH levels may be slightly altered among these women. However, the discrepancy compared to normocyclic PCOS patients is not clinically significant. Future studies in this field might help us shed more light in this particular group of women to determine whether they actually need closer follow-up.
